# Supplementary material for: High rate of critical coronary stenosis in comatose patients with Non-ST-elevation out-of-hospital cardiac arrest (NSTE-OHCA) undergoing therapeutic hypothermia—Experience from the HAnnover COoling REgistry (HACORE)
Source: PLoS One. 2021 May 4;16(5):e0251178. doi: 10.1371/journal.pone.0251178 (PMC8096113; doi:10.1371/journal.pone.0251178)
Supplement: S1 Table — (DOCX) [file pone.0251178.s001.docx]

**S1 Table: Characteristics regarding PCI of NSTE-OHCA and non-shockable patients**

| Demographics | NSTE  with PCI |  | NSTE  without PCI |  | p value | Non-shockable rhythm  with PCI | | Non-shockable rhythm  without PCI | | p value |
| --- | --- | --- | --- | --- | --- | --- | --- | --- | --- | --- |
| Number (%) | 118 | (35) | 219 | (65) |  | 65 | (33) | 135 | (63) |  |
| Age – years | 67±11 |  | 63±16 |  | 0.007 | 68±11 |  | 67±15 |  | 0,524 |
| Male sex, n (%) | 101 | (86) | 146 | (67) | <0.001 | 50 | (77) | 88 | (65) | 0.104 |
| Intrahospital survival(%) | 60 | (51) | 107 | (49) | 0.820 | 29 | (45) | 49 | (36) | 0.353 |
| Cardiovascular risk factors |  |  |  |  |  |  |  |  |  |  |
| Hypertension (%) | 74 | (63) | 117 | (53) | 0.108 | 41 | (63) | 69 | (51) | 0.130 |
| Diabetes (%) | 36 | (31) | 50 | (23) | 0.206 | 21 | (32) | 37 | (27) | 0.708 |
| Hyperlipidaemia (%) | 42 | (36) | 62 | (28) | 0.176 | 30 | (46) | 36 | (27) | 0.010 |
| Family history for CAD (%) | 11 | (9) | 14 | (6) | 0.384 | 3 | (5) | 3 | (2) | 0.393 |
| Smoking (%) | 38 | (32) | 49 | (22) | 0.052 | 20 | (31) | 27 | (20) | 0.110 |
| Previous comorbidities |  |  |  |  |  |  |  |  |  |  |
| CAD (%) | 33 | (28) | 61 | (28) | 1.000 | 19 | (29) | 35 | (26) | 0.615 |
| PCI (%) | 17 | (14) | 22 | (10) | 0.284 | 7 | (11) | 15 | (11) | 1.000 |
| CABG (%) | 13 | (11) | 33 | (15) | 0.324 | 6 | (9) | 15 | (11) | 0.808 |
| PAD (%) | 11 | (9) | 20 | (9) | 1.000 | 6 | (9) | 16 | (12) | 0.639 |
| TIA/stroke (%) | 13 | (11) | 29 | (13) | 0.608 | 10 | (15) | 19 | (14) | 0.832 |
| CKD (%) | 17 | (14) | 35 | (16) | 0.754 | 8 | (12) | 23 | (17) | 0.532 |
| chronic RRT (%) |  |  |  |  |  | 2 | (3) | 2 | (1) | 0.597 |
| Atrial fibrillation (%) | 35 | (30) | 52 | (24) | 0.243 | 15 | (23) | 24 | (18) | 0.446 |
| Pacemaker (%) | 7 | (6) | 6 | (3) | 0.233 | 3 | (5) | 2 | (1) | 0.332 |
| ICD (%) | 1 | (1) | 3 | (1) | 1.000 | 2 | (3) | 2 | (1) | 0.597 |
| COPD/ Asthma (%) | 17 | (14) | 28 | (13) | 0.738 | 11 | (17) | 22 | (16) | 1.000 |
| Characteristics of cardiac arrest |  |  |  |  |  |  |  |  |  |  |
| Witnessed arrest (%) | 100 | (85) | 159 | (73) | 0.014 | 50 | (77) | 92 | (68) | 0.245 |
| Bystander CPR (%) | 72 | (61) | 142 | (65) | 0.553 | 38 | (58) | 77 | (57) | 0.880 |
| Shockable Rhythm (%) | 80 | (68) | 91 | (42) | <0.001 | - | - | - | - | - |
| ST-segment elevation (%) | - | - | - | - | - | 27 | (42) | 7 | (5) | <0.001 |
| ROSC, min | 27±23 |  | 25±20 |  | 0.561 | 63±28 |  | 131±20 |  | 0,161 |
| Ongoing CPR at admission (%) | 13 | (11) | 26 | (12) | 0.860 | 12 | (18) | 17 | (13) | 0.293 |
| eCPR (%) | 6 | (5) | 7 | (3) | 0.392 | 5 | (8) | 5 | (4) | 0.300 |
| Baseline laboratory values |  |  |  |  |  |  |  |  |  |  |
| Lactate, mmol/l | 7.79±3.97 |  | 8.46±4.60 |  | 0,186 | 9.28±4.19 |  | 9.13±4.62 |  | 0,831 |
| pH | 7.16±0.17 |  | 7.13±0.18 |  | 0,103 | 7.09±0.19 |  | 7.08±0.18 |  | 0,845 |
| Creatinine, µmol/l | 137±102 |  | 145±130 |  | 0,568 | 146±112 |  | 169±159 |  | 0,305 |
| Urea nitrogen, mmol/l | 7.89±3.89 |  | 7.78±3.75 |  | 0,936 | 9.58±3.69 |  | 8.63±4.21 |  | 0,641 |
| Creatinkinase, U/l | 585±1756 |  | 403±962 |  | 0,302 | 615±1566 |  | 359±573 |  | 0,207 |
| hs-Troponin T, µg/l | 1337±8245 |  | 266±849 |  | 0,169 | 2315±10912 |  | 184±556 |  | 0,031 |
| NT-proBNP, ng/l | 732±2263 |  | 1023±3097 |  | 0,538 | 1107±2967 |  | 1152±3757 |  | 0,949 |
| Haemoglobin, g/dl | 13.04±2.38 |  | 12.54±2.87 |  | 0,261 | 12.93±2.74 |  | 11.97±3.15 |  | 0,103 |
| Leukocytes, *1000/µl | 15.78±8.39 |  | 14.73±7.63 |  | 0,43 | 15.43±6.34 |  | 15.15±7.65 |  | 0,846 |
| NSE- day 3, µg/l | 74(22-82) |  | 67(18-48) |  | 0,661 | 103(21-143) |  | 87(19-95) |  | 0,514 |
| S-100b- day 3, µg/l | 0.558(0.088-0.301) |  | 17.349(0.069-0.203) |  | 0,41 | 0.958(0.127-0.767) |  | 33,453(0.080-0.286) |  | 0,447 |

**CAD – coronary artery disease; CABG – coronary artery bypass graft; CKD – chronic kidney disease; COPD – chronic obstructive pulmonary disease; CPR – cardiopulmonary resuscitation; eCPR – ECMO-CPR; ECMO – extracorporeal membrane oxygenation; ICD – implantable cardioverter-defibrillator; PAD – peripheral artery disease; PCI – percutaneous coronary intervention; ROSC – return of spontaneous circulation; RRT – renal replacement therapy; TIA – transient ischemic attack.**
