# Supplementary material for: High rate of critical coronary stenosis in comatose patients with Non-ST-elevation out-of-hospital cardiac arrest (NSTE-OHCA) undergoing therapeutic hypothermia—Experience from the HAnnover COoling REgistry (HACORE)
Source: PLoS One. 2021 May 4;16(5):e0251178. doi: 10.1371/journal.pone.0251178 (PMC8096113; doi:10.1371/journal.pone.0251178)
Supplement: Attachment — Submitted filename: 20210309_Revision 1_HACORE ECG.docx [file pone.0251178.s002.docx]

Response to Editor Manuscript PONE-D-21-01798

We would like to thank the Editor for his/her constructive criticism and valuable input. We hope that the changes made to the manuscript and the responses detailed below, satisfactorily address the concerns raised.

*1. Please ensure that your manuscript meets PLOS ONE's style requirements, including those for file naming.*

The manuscript has been formatted according to the recommendations given prior to any responses to the reviewers.

*2. Please provide additional details regarding participant consent. In the ethics statement in the Methods and online submission information, please ensure that you have specified (1) whether consent was informed and (2) what type you obtained (for instance, written or verbal, and if verbal, how it was documented and witnessed). If your study included minors, state whether you obtained consent from parents or guardians. If the need for consent was waived by the ethics committee, please include this information.*

*If you are reporting a retrospective study of medical records or archived samples, please ensure that you have discussed whether all data were fully anonymized before you accessed them and/or whether the IRB or ethics committee waived the requirement for informed consent. If patients provided informed written consent to have data from their medical records used in research, please include this information.*

Written informed consent was obtained from legal guardians during the unconscious period and re-consented by survivors after gaining consciousness. Anonymized data were entered into the database. This statement is now included in the Methods section (lines 95-100) and the online submission from.

*3. We note that you have indicated that data from this study are available upon request. PLOS only allows data to be available upon request if there are legal or ethical restrictions on sharing data publicly. For information on unacceptable data access restrictions, please see http://journals.plos.org/plosone/s/data-availability#loc-unacceptable-data-access-restrictions.*

*In your revised cover letter, please address the following prompts:*

*a) If there are ethical or legal restrictions on sharing a de-identified data set, please explain them in detail (e.g., data contain potentially identifying or sensitive patient information) and who has imposed them (e.g., an ethics committee). Please also provide contact information for a data access committee, ethics committee, or other institutional body to which data requests may be sent.*

Regarding data availability there is a legal restriction as the patient sample includes potentially identifying patient information and specific dates. Contact information for the ethics committee is: [Ethikkommission@mh-hannover.de](mailto:Ethikkommission@mh-hannover.de). This has been included in the revised cover letter.

4. *We note that the grant information you provided in the ‘Funding Information’ and ‘Financial Disclosure’ sections do not match.*

*When you resubmit, please ensure that you provide the correct grant numbers for the awards you received for your study in the ‘Funding Information’ section.*

The only support was as stated in the funding section “This study was partly supported by the Deutsche Forschungsgemeinschaft (DFG), Klinische Forschergruppe (KFO) 311, TP1.” However, it was not possible to enter this during the initial submission.

5. *Thank you for stating the following in the Competing Interests section:*

*"AS received modest lecture fees from ZOLL Inc. regarding therapeutic hypothermia. All other authors have no conflict of interest to declare."*

*Please confirm that this does not alter your adherence to all PLOS ONE policies on sharing data and materials, by including the following statement: "This does not alter our adherence to PLOS ONE policies on sharing data and materials.” (as detailed online in our guide for authors http://journals.plos.org/plosone/s/competing-interests). If there are restrictions on sharing of data and/or materials, please state these. Please note that we cannot proceed with consideration of your article until this information has been declared.*

*Please include your updated Competing Interests statement in your cover letter; we will change the online submission form on your behalf.*

The statement has been included in the manuscript (lines 344-345) and in the cover letter.

6. *Please include captions for your Supporting Information files at the end of your manuscript, and update any in-text citations to match accordingly. Please see our Supporting Information guidelines for more information.*

The caption for S1 table has been added at the end of the manuscript (line 458).

7. *Your ethics statement should only appear in the Methods section of your manuscript. If your ethics statement is written in any section besides the Methods, please delete it from any other section.*

The ethics statement at the declaration section has been removed as requested.

8.*We noticed you have some minor occurrence of overlapping text with the following previous publication(s), which needs to be addressed:*

*- https://www.jacc.org/doi/full/10.1016/j.jcin.2018.06.022*

*The text that needs to be addressed involves sentences 1, 2, 8, 9, and 10 of the Introduction. Overlap also should be addressed in Paragraph 2, sentences 3 and 5 of the Discussion.*

*In your revision ensure you cite all your sources (including your own works), and quote or rephrase any duplicated text outside the methods section. Further consideration is dependent on these concerns being addressed.*

The sentences in the introduction section have been modified (lines 74-84), sentences 8-10 could have been transferred to the methods section if required to exclude duplication.

The sentences in the discussion have been rephrased as indicated (lines 241-244 & 247-250).
Response to Reviewer #1 Manuscript PONE-D-21-01798

We would like to thank the reviewer for his/her constructive criticism and valuable input. We hope that the changes made to the manuscript and the responses detailed below, satisfactorily address the concerns raised.

*Introduction*

*- your study is focused on post-ROSC ECG and PCI, but you have focused your introduction more on brain and hypothermia. I suggest to re-balance your introduction.*

As suggested by the reviewer, we have rewritten the introduction now focusing primarily on post-ROSC-ECG and indication for PCI (lines 61-66).

*- moreover, especially in the first part of the introduction, many sentence require a reference (e.g. line 58, line 62 and line 64-65).*

The reference to recent AHA ACLS guidelines has been added early on. Most of the other requirements for references are not required anymore as the sentences under concern have been deleted as requested above.

*Methods*

*- please, specify when you chose, in your hospital, to use ECMO rather than Impella*

Microaxial pumps were used primarily in isolated left-ventricular failure whereas ECMO was used in refractory arrest or biventricular failure. This has now been added to the patient population section (lines 112-116).

*Results:*

*- line 163: please, change ECG in rhythm evaluation. ECG here is misleading*

The wording has been changed as suggested (line 166).

*- line 168 and line 191: I believe that "when trying to identify potential discriminating factors" should be changed in a more simply "Comparing PCI and non-PCI group..."*

The wording has been changed as suggested (lines 171).

*- line 176 and line 194: please, specify which are the inclusion criteria of COACT-like and HYPERON-like patients to improve clarity for the readers. Moreover, you should pre-specify these sub-group analysis in the methods*

In the revised version of the manuscript we focused on the COACT trial as it is a trial selectively addressing PCI in NSTE-OHCA patients and we have skipped the reference to the HYPERION trial as also suggested by reviewer #2. Inclusion and major exclusion criteria have been added at the suggested part of the text including the respective reference (lines 180-181). The respective sub-group analysis has now been pre-specified in the Methods section (lines 140-144).

*Discussion:*

*- in general I believe that the discussion should be deeply revised for these reasons:*

*-- you study is interesting, however it is a observational study and not a RCT, so you have all the typical limitation of the observational studies, including selection bias and a limited sample size. Therefore I believe you cannot state (directly or indirectly) that your study can provide more useful results than a RCT as COACT.*

We have changed the discussion section with regard to this matter. Our intention was not to imply that an observational study provides better results than a RCT. However, observational studies can provide useful information if they indicate that a RCT had been very selective. To this regard, the COACT criteria applied to less than one third of our observational cohort. In other words, every day’s clinical life is much more complex and patients differ largely from the cohort selectively investigated in the trial. Therefore, we believe it is warranted to raise some caution regarding extrapolation of study results into a much more heterogeneous and eventually sicker patient population in every day clinical routine. Following publication of COACT, there was an urge by colleagues that it might generally not be necessary to perform coronary angiography in resuscitated patients if they do not show ST-elevations. However, our registry shows that most patients in clinical routine were not represented by the trial population. Therefore, we tried to find some further indicator to guide patient flow regarding potential beneficiaries from coronary angiography. Following our experience, there is a higher rate of PCI in NSTE-OHCA patients with shockable rhythm; on the contrary, PCI rate and survival in non-shockable rhythms are very low. Therefore, combination of primary rhythm and absence of ST-segment elevations might be a useful indicator to guide patient flow.

*The same when comparing Minnesota study with COACT, Minnesota is observational, COACT RCT. I suggest to focus on those sub-groups of patients excluded from the COACT, for example the patients with shock.*

*-- please, considering all above, mitigate your conclusions*

We thank the reviewer for pointing out this important point. However, with all due respect to the reviewer, given the data in our registry it rather appears that the patients in COACT represent a subgroup of the patients being admitted in everyday clinical routine. We never intended to question the trial results; we rather observed a similar outcome in COACT-like patients in HACORE (lines 293-294). Nevertheless, the majority of our patients would have been excluded from the trial mainly due to presence of haemodynamic instability. Considering that these patients did not have STE, all had been resuscitated, and all were in shock, 42% survival is not too bad a result (lines 271-280).

*-- I believe that in the discussion you should focus also on the difference regarding survival between shockable and non-shockable (due to different etiology?)*

We agree with the reviewer that the presence or absence of a shockable rhythm might indicate a potential different etiology of cardiac arrest. Shockable might be more likely be indicative for an ischaemic cause, whereas non-shockable might be attributed to anoxic causes. Indeed, baseline characteristics do support such a hypothesis depicting a significantly higher number of chronic pulmonary disease in patients with non-shockable rhythm and a higher proportion of ST-elevation in shockable rhythm. Patients with non-shockable rhythm had worse conditions of resuscitation indicated by less witnessed arrest and bystander CPR potentially contributing to higher admission lactate and NSE levels (lines 312-321).

*-- you skipped in the discussion an issue that I believe it is very important. About 20% of the STE-patients were not treated with a PCI, this is consistent with previous data in literature and should be stressed. Moreover, considering recent data regarding the key role of timing from ROSC to first ECG acquisition to decrease the percentage of false-positive ECG (JAMA Netw Open. 2021;4(1):e2032875. doi:10.1001/ jamanetworkopen.2020.32875), please consider to comment your results in light of this recent evidence*

We agree with the reviewer on this important point, which has been added to the discussion section. Prior to the cited analysis we intended to perform a 12-channel ECG as rapidly as possible after ROSC (lines 229-235).

*- line 254: "had had" is a typo*

We apologize for that, the typo has been corrected; a past perfect does not seem to be necessary.

*Limitations*

*- please, consider your limited sample size as a limitation. Moreover, your study is observational, therefore this should be recognized in the limitations*

This has been done as requested (lines 327-329).

*- your registry consider only patients in whom hypothermia is performed. This is an important limitation and selection bias and should be highlighted*

With all due respect to the reviewer, we do not believe that strong adherence to guideline recommendations is an important limitation. Our protocol and registry ensure that there is a strict approach towards post-arrest patients in our centre. We just want to confirm to the reviewer that there are no comatose OHCA patients in our hospital with presumed cardiac cause of arrest who are treated by normothermia or no TTM at all. This aspect regarding the setting has been highlighted in detail in the introduction section (lines 82-84).

Response to Reviewer #2 Manuscript PONE-D-21-01798

We would like to thank the reviewer for his/her constructive criticism and valuable input. We hope that the changes made to the manuscript and the responses detailed below, satisfactorily address the concerns raised.

*The conclusions expressed in the abstract and in the full paper are slightly different. In the full paper the conclusion is that NSTEMI non-shockable rhythm do not deserve angiography (since just 22% had PCI), while in the abstract is stated that patients with NSTEMI shockable rhythm deserve immediate angiography (since the authors compare the STEMI shockable survival with NSTEMI shockable survival). So if the real conclusions are the ones expressed in the abstract, I think that some information are missing, in particular there is no data about survival in the subgroups NSTEMI shockable PCI vs NSTEMI shockable non PCI. The 60% of survival in NSTEMI shockable patients, which is similar to the STEMI shockable ones (61%), refers to all the NSTEMI shockable (no distinction between PCI vs no PCI), which may be crucial in recommending angiography in this population.*

We thank the reviewer for pointing out the differing wording which we have corrected in the revised version. However, there is one point in the wording which we would like to strengthen: we used the expression of NSTE-OHCA instead of NSTEMI, because it is very difficult and in many cases impossible after resuscitation to clarify by laboratory parameters alone (e.g. troponin) whether there is an myocardial infarction responsible for release of troponin and the “NSTEMI” is cause of arrest or whether the release of myocardial biomarkers is the consequence of arrest and resuscitation efforts. While patients with NSTEMI with subsequent arrest might have the chance to profit from coronary angiography it is very unlikely that patients with cardiac arrest not related to coronary ischaemia will have a benefit. We have elaborated that problem in the revised introduction section.

As requested, we modified the abstract to be in line with the final conclusion.

*Moreover, it's not clear the purpose in citing the HYPERION trial (lines 293-294) in which there is no mention about the link between PCI-rate and in-hospital mortality rate*

We agree with the reviewer that the focus of the HYPERION trial is on therapeutic hypothermia rather than on ECG and PCI and we have, therefore, removed the respective sentences.
