# Supplementary material for: High rate of critical coronary stenosis in comatose patients with Non-ST-elevation out-of-hospital cardiac arrest (NSTE-OHCA) undergoing therapeutic hypothermia—Experience from the HAnnover COoling REgistry (HACORE)
Source: PLoS One. 2021 May 4;16(5):e0251178. doi: 10.1371/journal.pone.0251178 (PMC8096113; doi:10.1371/journal.pone.0251178)
Supplement: Attachment — Submitted filename: 20210416_Revision 2_HACORE ECG.docx [file pone.0251178.s003.docx]

Response to Editor Manuscript PONE-D-21-01798R1

We would like to thank the Editor for his/her constructive criticism and valuable input. We hope that the changes made to the manuscript and the responses detailed below, satisfactorily address the concerns raised.

*Thank you very much for having addressed the majority of the reviewers' comments. However some uncovered issues still remain as highlighted by the reviewers. The first is concerning your enrolling only patients who underwent to therapeutic hypothermia. I have no doubt that you apply therapeutic hypothermia according to guidelines. However, by doing so you did not consider those patients resuscitated from an OHCA and not matching with the hypothermia indications after ROSC (e.g. the awake patients). So my advice for you is either to acknowledge that as a limitation or to state clearly in the title that you are referring only to hypothermic patients.*

As suggested by the editor and the reviewer we do now clearly state that fact in the limitations section and have adopted the title accordingly (pages 1&18).

*The second concern is about the comparison in terms of survival between NSTE patients receiving or not a PCI. The p value required by reviewer#2 is of pivotal importance because some of your statements are grounded on that.*

As we explain in the point-by-point response there was a rationale not to compare those populations directly, because they appear to be very different regarding the principal cause of arrest (ischemic vs non-ischemic). Nevertheless, as it seems to be a very important point to the reviewer and we acknowledge his/her positive and constructive feedback during the process of revision, we have now calculated the p-value and reported it in the main text (page 13).

Response to Reviewer #1 Manuscript PONE-D-21-01798R1

We would like to thank the reviewer for his/her constructive criticism and valuable input. We hope that the changes made to the manuscript and the responses detailed below, satisfactorily address the concerns raised.

*Dear Authors, thank you so much for addressing the majority of my concerns.*

*I have few minor concerns:*

*- line 55: you stated that "arrhythmias" is a cause of OHCA of cardiac etiology. However, I believe that arrhythmias are the epiphenomen of an underlying heart disease. Or do you mean "primary arrhythmias" such as in LQTS, SdB and so on?*

Indeed, arrhythmias are in most cases considered an epiphenomenon of underlying heart disease as stated by the reviewer. We have stated this now more clearly as suggested (page 3).

*- line 58 and 61: "while urgent"... "while urgent", please rephrase*

We have modified the first sentence in order to remove the similar start of subsequent sentences as suggested by the reviewer (page 3).

*- line 69-70 "uninterrupted phase of therapeutic hypotermia following ICU admission": there are many demonstrations that coronary angiography can be performed during hypotermia, therefore the decision to perform coronary angiography before ICU admission cannot be based on this justification*

We do agree with the reviewer that it is technically feasible to perform coronary angiography during hypothermia. Nevertheless, we wanted to express that we intend to provide consecutive intensive care in general without requiring patients to be transported to other departments for diagnostic procedures such as coronary angiography or computed tomography. As described in Table 1, quite a number of patients are either on renal replacement therapy and/or mechanical circulatory support and unnecessary transports are kept to a minimum. We hope that the revised wording is clearer to understand and not as misleading as the previous one (page 3-4).

*Methods:*

*- line 106: as outlined in my previous revision, I believe that the fact that you included only patients who undergone to hypotermia has to be addressed as a limitation. In fact, following the guidelines, hypotermia is for unconscious patients after ROSC, therefore you excluded all the conscious patients after ROSC, and it is a limitation*

We now clarified the fact that all patients were still comatose and receiving hypothermia in the title, the patient population, and the limitations section. (pages 1, 5 & 18).

*Results:*

*- line 155: you state that 116 OHCA patients required mechanical support for cardiogenic shock (Table 1). However, in Table 1 if you add the patients with eCPR, those with Impella and those with vaECMO they are more than 116. Why?*

The overall number of patients on mechanical support was 116 as stated in the text. E.g. all eCPR patients are also included in the vaECMO line and several patients received vaECMO plus Impella (n=37 in total), which attributes to the higher number when adding eCPR, Impella and vaECMO. This has now been explained in more detail (page 7).

*Discussion*

*- line 226: change "during first rhythm control" in "shockable presenting rhythm"*

This has been changed as suggested (page 13).

*- line 290-295: I'm aware that the COACT had selective inclusion criteria, but the trial was designed in that way and it is speculative to argue that someone wants to extrapolate the results of a trial (which has, by definition, inclusion and exclusion criteria) to the general population. It is like you argue to extrapolate the results of a trial about the use of Ticagrelor in STEMI patients to all the patients with cardiac ischemic disease.*

We have rephrased that paragraph and now exclude the speculative part just stating that the overall NSTE-OHCA cohort included a large proportion of shock patients (page 16).

*- line 307-310: I believe that here you introduce a very important point that should be more highlighted, as the importance of a stop in Emergency Department to exclude non-cardiac cause, as recommended by 2015 ESC Guidelines on ventricular arrhythmias.*

We thank the reviewer for this suggestion and have quoted the 2015 ESC guideline as suggested (page 17).

Response to Reviewer #2 Manuscript PONE-D-21-01798

We would like to thank the reviewer for his/her constructive criticism and valuable input. We hope that the changes made to the manuscript and the responses detailed below, satisfactorily address the concerns raised.

*Why should routine early PCI be performed on patients with primary shockable rhythms during NSTE-OHCA if it is stated that the difference of survival between NSTE-OHCA patients with primarily shockable rhythm receiving PCI (56%) and those not requiring PCI (64%) was minor (p=??)? It seems that by performing PCI in these patients there is no survival benefit.*

We acknowledge this important remark by the reviewer, but due to the observational and non-interventional registry design, we believe that we should not draw this conclusion. We would be allowed to conclude like that, if we had identified NSTE-OHCA patients with primarily shockable rhythms who had relevant coronary stenosis and had them randomized to PCI vs conservative treatment. In our manuscript (and the registry), however, we have the group with relevant coronary stenosis receiving PCI (coronary artery disease as most probable cause of arrest) and the group without coronary stenosis treated conservatively (underlying heart disease with arrhythmic cause of arrest). Therefore, neither can we state that performing PCI provides a benefit nor that “performing PCI in these patients is without survival benefit”. We just compare to very different groups of patients.

Our primary intention was to describe the pattern of underlying disease in a cohort that received routine angiography. To enhance the discussion about the impact on outcome, we have now added the data on good neurological outcome as assessed by cerebral performance category ≤2 in addition to hospital survival and discharge in Figure 3, which now illustrates even more that initial rhythm seems to be more important for outcome than ST-segment elevation. Still, we cannot state that NSTE-OHCA patients receiving PCI have better outcomes than those not receiving PCI. However, we can illustrate that it is not the absence or presence of ST-elevations that is predictive for outcome, but rather the primary rhythm that is. As these patients’ potential outcome is not as futile as many think, we want to encourage the discussion about providing similar care regarding treatment of cardiac ischaemia as it is provided in STE-OHCA patients. This has been added to the discussion on page 16.

Nevertheless, the requested p-value has been added to the sentence on page 13.
